# Specific Treatment of Diphtheria

**Published:** 1884-09

**Authors:** 


### Specific Treatment of Diphtheria by the Combustion of a Mixture of Oil of Turpentine and Coal Tar, or by the Oil of Turpentine Alone.

This specific treatment of diphtheria, which has given the author the best results in croup also, consists in igniting, in the sick chamber, a mixture of coal tar and oil of turpentine, in the average proportion of 200 gram. of the first to 80 gram. of the second (℥vi to ℥iiss), or even the oil of turpentine alone in default of the coal tar, for the Norwegian tar provokes cough.

It produces an intense smoke which has the power of softening and disintegrating the pseudo-membranes, which are afterward expelled as catarrhal secretions.

The amelioration is rapidly obtained, but it is necessary to prolong the fumigations more or less time according to the gravity of the cases.

Dr. Delthil considers this a heroic method at the start of the disease, but in croup, when the physician is called late, when there is but the supreme resource of tracheotomy left, it is yet of advantage to use the fumigations in connection with the operation.

He reports a case of tracheotomy, in a child who had a general diphtheria and whose bronchial obstruction had been ascertained by other physicians. After the operation, fumigations were made during many hours, which had the effect to fluidify and eliminate the bronchial and tracheal pseudo-membranes.

The combustion of the turpentine and coal tar produces such a profusion of carburets and well divided carbon in the chamber, that the light is dimmed, yet, the patient and the assistants respire well in this atmosphere which provokes no cough.—*Ex.*

---

